# Spectral Characteristics and Functional Responses of Phospholipid Bilayers in the Terahertz Band

**DOI:** 10.3390/ijms24087111

**Published:** 2023-04-12

**Authors:** Yanyun Lin, Xingjuan Wu, Kaicheng Wang, Sen Shang, Yubin Gong, Hongwei Zhao, Dai Wu, Peng Zhang, Xiaoyun Lu

**Affiliations:** 1 Key Laboratory of Biomedical Information Engineering of Ministry of Education, School of Life Science and Technology, Xi’an Jiaotong University, Xi’an 710049, China; 2 Medico-Engineering Cooperation on Applied Medicine Research Center, University of Electronic Science and Technology of China, Chengdu 610054, China; 3 Shanghai Advanced Research Institute, Chinese Academy of Sciences, Shanghai 201204, China; 4 Institute of Applied Electronics, China Academy of Engineering Physics, Mianyang 621900, China

**Keywords:** phospholipid bilayers, THz absorption spectra, cell membrane fluidity, phagocytosis

## Abstract

Understanding the vibrational information encoded within the terahertz (THz) spectrum of biomolecules is critical for guiding the exploration of its functional responses to specific THz radiation wavelengths. This study investigated several important phospholipid components of biological membranes—distearoyl phosphatidylethanolamine (DSPE), dipalmitoyl phosphatidylcholine (DPPC), sphingosine phosphorylcholine (SPH), and lecithin bilayer—using THz time-domain spectroscopy. We observed similar spectral patterns for DPPC, SPH, and the lecithin bilayer, all of which contain the choline group as the hydrophilic head. Notably, the spectrum of DSPE, which has an ethanolamine head group, was different. Interestingly, density functional theory calculations confirmed that the absorption peak common to DSPE and DPPC at approximately 3.0 THz originated from a collective vibration of their similar hydrophobic tails. Accordingly, the cell membrane fluidity of RAW264.7 macrophages with irradiation at 3.1 THz was significantly enhanced, leading to improved phagocytosis. Our results highlight the importance of the spectral characteristics of the phospholipid bilayers when studying their functional responses in the THz band and suggest that irradiation at 3.1 THz is a potential non-invasive strategy to increase the fluidity of phospholipid bilayers for biomedical applications such as immune activation or drug administration.

## 1. Introduction

Terahertz (THz) waves, with frequencies ranging from 0.1 to 10 THz, belong to the transitional zone between electronics (10^9^ Hz) and photonics (over 10^13^ Hz). These frequencies correspond to photon energies in the 0.41–41 meV range (i.e., in the order of one-millionth of those of X-rays), indicating the non-ionizing property and excellent biological safety of THz radiation. The THz band spans the frequencies of the rotations and delocalized vibrations of most biomolecules, and hence, the THz spectra can provide information on biomolecular structures and interactions other than the characteristic localized vibration information offered by Fourier transform infrared and Raman spectroscopy. In addition, some studies have suggested that THz radiation may have various bioeffects mediated by the phonon or intermolecular modes of biomolecules [1,2,3]. Due to these properties, the spectral characteristics of biomolecules in the THz band and their functional responses have gradually become a focus of frontier scientific investigation in the field of biomedical optics.

Currently, THz time-domain spectroscopy (THz-TDS) technology is widely used to identify characteristic fingerprint spectra of various biomolecules such as nucleotides [4], amino acids [5], carbohydrates [6], and neurotransmitters [7]. However, studies on the THz spectra of phospholipids remain limited. Phospholipids contain a “hydrophilic head” comprising a substituent group linked by phosphoric acid and one or more “hydrophobic tails” consisting of fatty acid chains. In an aqueous solution, the hydrophobic tails align to face each other, whereas the “hydrophilic heads” are exposed outward to form a phospholipid bilayer, which is the primary component of cell membranes, mitochondrial membranes, and endoplasmic reticulum membranes [8]. Many different phospholipid bilayers occur in biological membranes; phosphatidylethanolamines, phosphatidylcholines, and sphingomyelin bilayers are the most common types [9]. In a recent study, Andachi et al. revealed that the absorption coefficient of a lipid bilayer of 1,2-dimyristoyl-sn-glycero-3-phosphocholine (DMPC) in the low-frequency THz band decreased at lower temperatures. This phenomenon was positively correlated with the degree of DMPC bilayer hydration at a specific frequency, implying that the presence of water molecules affected the intermolecular interactions of DMPC [10]. In a subsequent study by the same group, weak absorption peaks of a 1,2-dimyristoyl-sn-glycero-3-phosphoryl-3-rac-glycerol (DMPG) bilayer at 1.08 and 2.10 THz were observed in its crystalline phase, whereas no characteristic absorption peaks were found in the gel phase. The absorption peak of the DMPG bilayer blue-shifted, and its peak absorption coefficient increased with decreasing temperature [11]. However, thus far, no robust characteristic phospholipid absorption peaks have been reported in the low-frequency THz band. This strongly suggests that the THz detection range for the bilayer should be broadened.

Liposomes and cell membranes are classic artificial and natural models for the analysis of the functional responses of phospholipid bilayers under THz radiation. Ramundo-Orlando et al. used 0.13- and 0.15-THz waves to irradiate liposomes composed of complex phospholipids at a power density of 6.2 mW/cm^2^. Interestingly, irradiation using either frequency band at a repetition frequency of 7 Hz resulted in improved permeability of the liposomes, whereas no effects were observed at a repetition frequency of 5 or 10 Hz [12]. In addition, a series of studies revealed that THz radiation with specific parameters could cause morphological changes as well as repairable or even irreparable perforations in the cell membranes of different cell types [13,14,15]. However, other studies have reported that THz radiation does not alter the morphologies and functions of cell membranes [16,17,18]. This contrary conclusion may be a result of the isolated study of the spectral characteristics and functional responses of phospholipid bilayers in the THz band. The characteristic absorption peaks of the phospholipid bilayers obtained from their spectroscopic studies were not fully utilized to direct the selection of the THz frequencies most likely to induce bioeffects, resulting in insignificant or negative responses. To summarize, although the literature presents some contradictions, existing evidence indicates that THz radiation may enhance the fluidity of the phospholipid bilayer, leading to improved liposome permeability, altered cell morphology, and repairable or irreparable perforation of the cell membranes.

In this study, four of the most common phospholipid bilayers in biological membranes—distearoyl phosphatidylethanolamine (DSPE), dipalmitoyl phosphatidylcholine (DPPC), sphingosine phosphorylcholine (SPH), and lecithin bilayer—were selected because of their subtle but key structural differences. We investigated the absorption spectra of these phospholipid bilayers via THz-TDS in the range of 0.5–4.0 THz. Furthermore, the effects of a temperature reduction from 293 to 83 K on the THz absorption spectra of the DSPE and DPPC bilayers were investigated. The density functional theory (DFT) calculations of the spectroscopic bands of the DSPE and DPPC bilayer revealed that their shared characteristic absorption peak at approximately 3.0 THz could be attributed to the intrinsic collective vibrations of the similar long hydrocarbon chains of their “hydrophobic tails”. Therefore, the 3.1-THz band was used to explore whether THz radiation could influence the cell membrane fluidity of RAW264.7 macrophages and the associated phagocytosis of RAW264.7 macrophages for micron-sized particles.

## 2. Results

### 2.1. THz Absorption Spectra of Phospholipid Bilayer

The primary variations between the different phospholipids lie in the length and/or saturation of the fatty acid chains and in the types of substituent groups linked by phosphoric acid. As shown in Figure S1a,b, the “hydrophobic tails” of DSPE and DPPC are saturated fatty acids, whereas the “hydrophilic head” of the former is an ethanolamine group and that of the latter is a choline group. SPH is abundant in the human nervous system. Its “hydrophobic tail” is an unsaturated fatty acid, whereas it has the same “hydrophilic head” as that of DPPC (Figure S1c). In contrast to the above-mentioned three phospholipids, lecithin is a phosphatidylcholine complex with an undefined fatty acid chain length and number of carbon–carbon double bonds. Thus, these four phospholipids have subtle but key structural differences. Since they are also present in biological membranes in relatively high amounts, they were considered as suitable models for the study of the THz absorption spectra of phospholipid bilayers.

**Figure 1:**
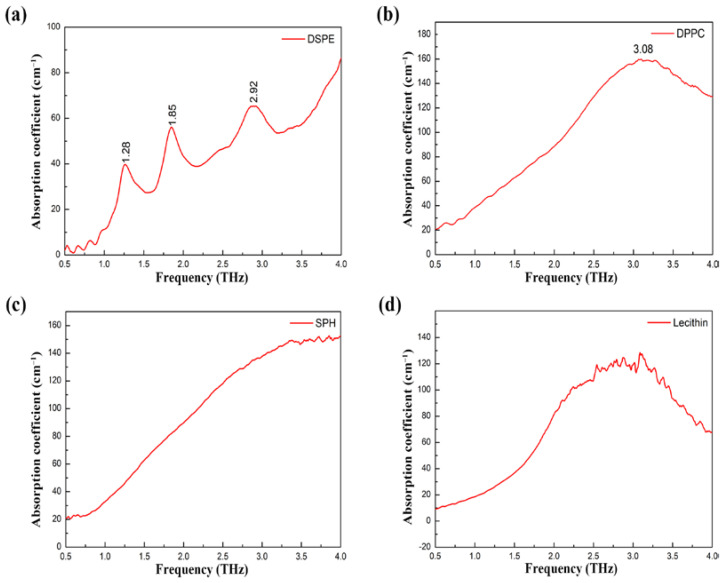
The THz absorption spectra of the (**a**) DSPE, (**b**) DPPC, (**c**) SPH, and (**d**) lecithin bilayers in the range of 0.5–4.0 THz at 293 K.

Cyclic olefin copolymer (COC) is an amorphous transparent polymer with a cyclic olefin structure that is formed by polymerizing bicycloheptene and ethylene. COC does not interact with phospholipid bilayers, and its absorption coefficient at 0.5–4.5 THz is lower than 2 cm^−1^ (Figure S2). Therefore, in this study, COC was employed as an ideal dilutant to improve the poor signal-to-noise ratio resulting from the strong phospholipid THz absorptions. The DSPE bilayer exhibited three characteristic absorption peaks, located at 1.28, 1.85, and 2.92 THz (Figure 1a). In contrast, the DPPC bilayer exhibited only a broad absorption peak at approximately 3.08 THz (Figure 1b). Although a characteristic absorption peak at approximately 3.0 THz was not observed for SPH, the SPH absorption coefficient no longer increased with a frequency exceeding 3.0 THz (Figure 1c). The broad character of its THz spectrum may be a result of the relatively low crystallinity of SPH, leading to the apparent obliteration of its characteristic absorption peak at 3.0 THz. The absorption spectrum of lecithin was similar to that of DPPC, indicating that the fatty acid chain length and the number of carbon–carbon double bonds had no significant influence on the THz absorption spectra of the phospholipid bilayers (Figure 1d). Overall, the THz absorption spectra of the DPPC, SPH, and lecithin bilayers exhibited the same pattern, which was remarkably distinct from that of DSPE. This is because for each of the first three phospholipids, the “hydrophilic head” is a choline group, whereas that of DSPE is an ethanolamine group. In other words, the resemblance between the chemical structures of the “hydrophilic head” was observed as similar molecular vibrational modes and THz absorption spectra. Hence, the DSPE and DPPC bilayers (the latter being representative of DPPC, SPH, and lecithin bilayers) were utilized in subsequent experiments.

### 2.2. Effects of Temperature on DSPE and DPPC THz Absorption Spectra

**Figure 2:**
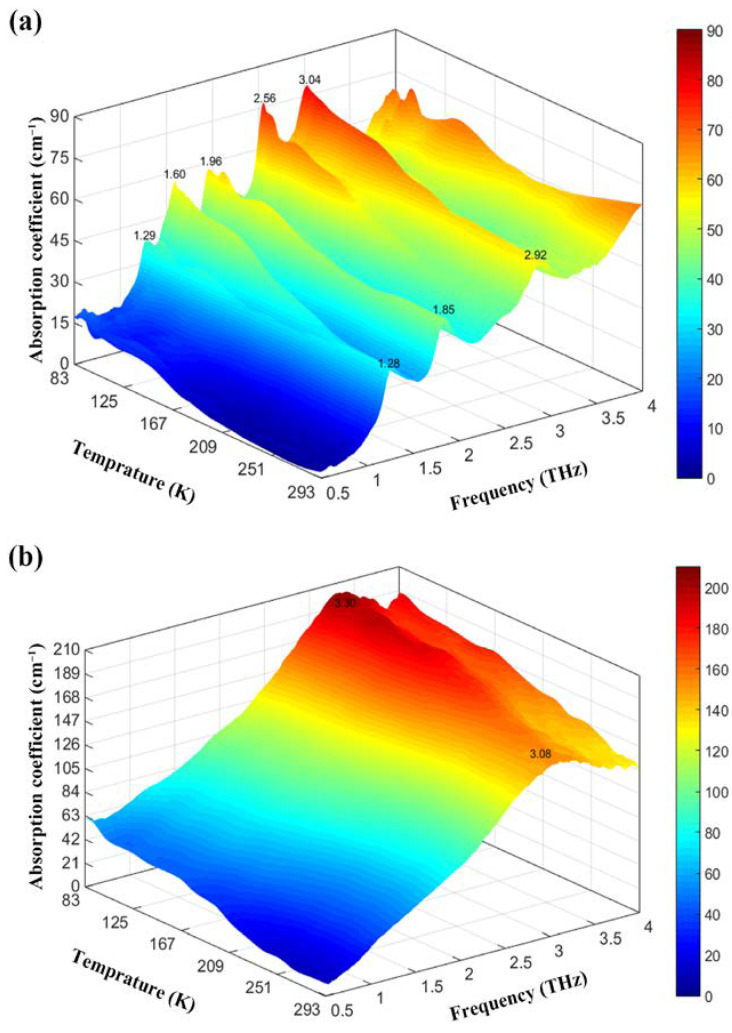
The THz absorption spectra of the (**a**) DSPE and (**b**) DPPC bilayer in the range of 0.5–4.0 THz at 83–293 K. The color scale and the vertical axis indicate the magnitude of the absorption coefficient.

In addition to focusing our research on the THz absorption spectra of different phospholipid bilayers, we explored the effects of temperature on the absorption coefficients of the DSPE and DPPC bilayers (Figure 2). At 293 K, DSPE displayed three characteristic absorption peaks at 1.28, 1.85, and 2.92 THz. As the temperature decreased, the absorption coefficient of DSPE bilayer gradually increased. At approximately 250 K, a new characteristic absorption peak emerged at approximately 1.5 THz. As the temperature decreased further to approximately 170 K, a new characteristic absorption peak appeared at approximately 2.5 THz. These new characteristic absorption peaks became significant as the temperature decreased even further. At 83 K, the DSPE bilayer exhibited eight characteristic absorption peaks including five intense peaks at 1.29, 1.60, 1.96, 2.56, and 3.04 THz and three relatively weak peaks at 2.23, 2.73, and 3.39 THz (Figure 2a). Notably, at low temperatures, the THz-TDS results revealed the molecular vibrational and rotational characteristics of the DSPE bilayer that were obscured by the intense molecular thermal motion at higher temperatures. Another notable phenomenon observed in this study was that the characteristic THz absorption peaks of the DSPE bilayer were blue-shifted as the temperature decreased. This result is consistent with the conclusions of other studies [11]. In contrast to DSPE, the DPPC bilayer did not display any new absorption peaks when the temperature was lowered. As the temperature decreased, the absorption coefficient of the DPPC bilayer at 3.08 THz increased, and this characteristic absorption peak was also blue-shifted (Figure 2b).

### 2.3. Common Absorption Peaks and Quantum Chemical Calculations of DSPE, DPPC and SPH

Based on the spectroscopic results described above, we concluded that the “hydrophilic head” of the phospholipid bilayer was the most critical factor in determining its spectral characteristics in the THz band, whereas the influences of the chain length of the fatty acids and the number of carbon–carbon double bonds were extremely minor. Interestingly, despite their different “hydrophilic heads”, the DSPE and DPPC bilayers exhibited a common absorption peak in the vicinity of 3.0 THz at 83–293 K (Figure 1 and Figure 2), which suggested that this particular peak could be assigned to a collective vibration of their similar phospholipid skeleton. To verify this hypothesis, molecular vibrational modes corresponding to the THz absorption spectra of DSPE, DPPC, and SPH were calculated based on DFT.

**Figure 3:**
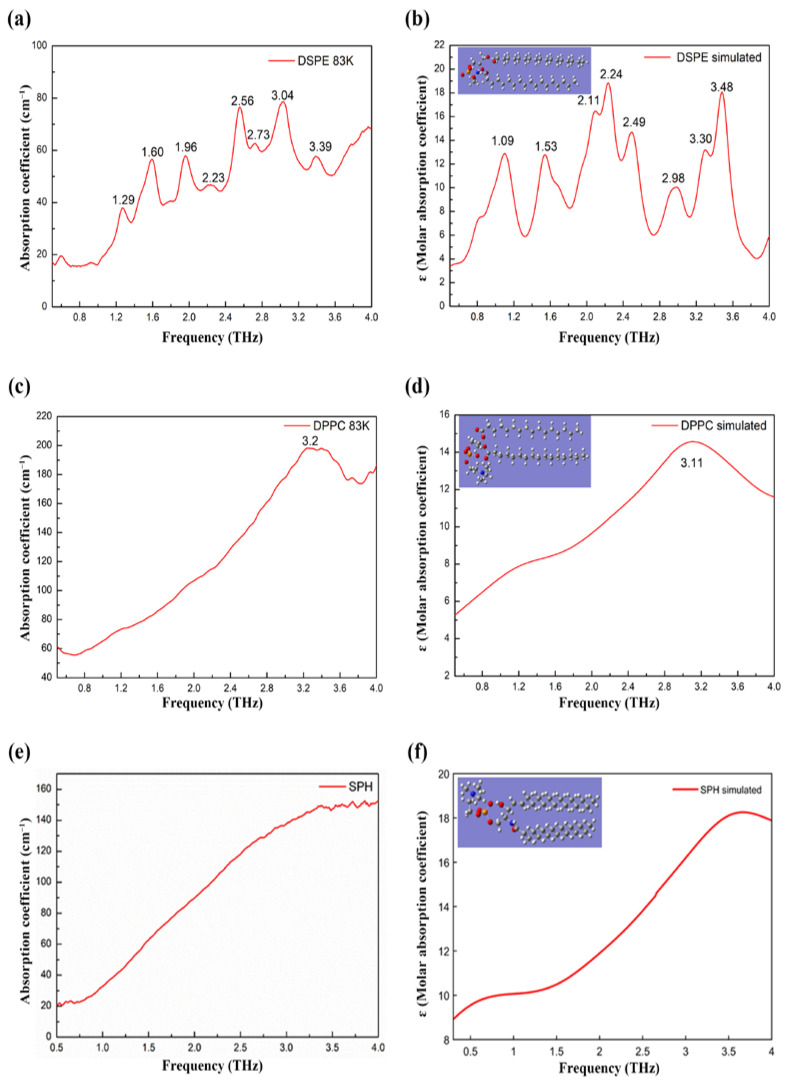
The experimentally measured and theoretically calculated THz absorption spectra of DSPE, DPPC, and SPH. The experimentally measured THz absorption spectra of the (**a**) DSPE, (**c**) DPPC and (**e**) SPH bilayers at 83 K. The theoretically calculated THz absorption spectra of (**b**) DSPE, (**d**) DPPC, and (**f**) SPH. The insets in (**b**,**d**,**f**) show the predicted molecular structures of DSPE, DPPC and SPH, respectively, following geometry optimization.

Quantum chemical calculation is an effective instrument for unraveling the intermolecular and intramolecular vibration information encoded in the THz absorption spectra. The theoretically calculated THz absorption spectra of various small molecules such as monosaccharides [6] and neurotransmitters [7] have been shown to be in excellent agreement with the corresponding experimentally measured spectra. However, the results of quantum chemical calculations for biomacromolecules such as phospholipids with more than 200 atoms have not been reported. Given the relatively straightforward interactions between H_2_O clusters (primarily the vibrations of hydrogen bonds and oxygen–hydrogen bonds), first, we selected 50 H_2_O clusters (150 atoms) to perform quantum chemical calculations based on DFT using the Gaussian 09 package at the B3LYP/3-21G level of theory. As shown in Figure S3, the theoretically calculated THz absorption spectrum of 50 H_2_O clusters was reasonably consistent with the experimentally measured spectrum, in which the absorption coefficient increased with frequency. This result implies a degree of reliability for this type of quantum chemical calculation for biomacromolecules with hundreds of atoms. The vibrational frequencies of DSPE, DPPC, and SPH were calculated using the same environmental parameters. The theoretically calculated absorption peaks of DSPE at 1.53, 2.11, 2.24, 2.98, and 3.48 THz corresponded to the absorption peaks at 1.29, 1.96, 2.23, 2.73, and 3.39 THz in the experimentally measured spectrum (Figure 3a,b). The theoretically calculated absorption peak of DPPC at 3.11 THz corresponded to the absorption peak at 3.2 THz in the experimentally measured spectrum (Figure 3c,d). The theoretically calculated absorption peak at approximately 3.0 THz was not observed for SPH, but the absorption coefficient no longer increased with a frequency exceeding 3.5 THz, which corresponded to the experimentally measured spectrum (Figure 3e,f). All of the vibrational modes resulting from the DFT calculation are shown in Table S1. The vibrational modes of DSPE at 2.98 THz and DPPC at 3.11 THz were both identified as collective vibrations, primarily confined to the similar long hydrocarbon chains of their “hydrophobic tails”. The above described results were consistent with the findings of Hielscher and Hellwig in 2010. They revealed that asolectin, cardiolipin, phosphatidylcholine, deuterated D22-phosphatidylcholine, and deuterated D35-phosphatidylcholine had a common absorption peak at around 3 THz. They reckoned that this signal mainly originated from the molecular breathing of phospholipid skeletons [19].

### 2.4. Enhanced Cell Membrane Fluidity of RAW264.7 Macrophages with Irradiation at 3.1 THz

**Figure 4:**
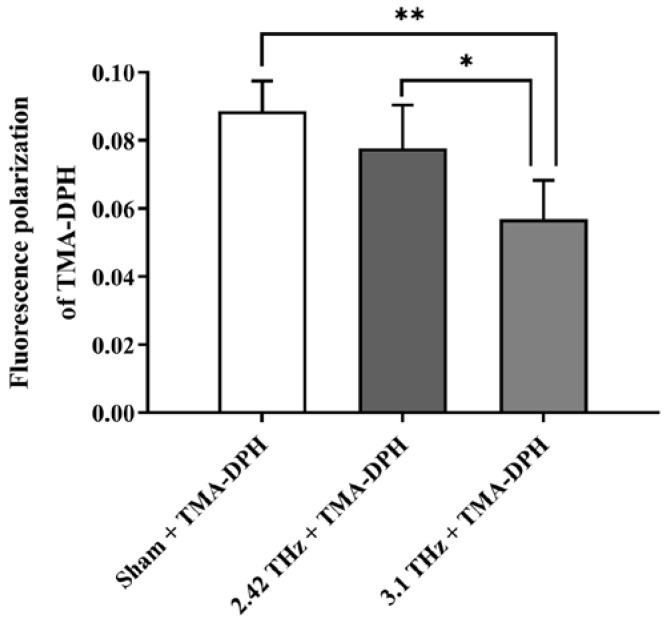
The effect of different frequencies of THz radiation on the cell membrane fluidity of RAW264.7 macrophages; * *p* < 0.05, ** *p* < 0.01.

The fluidity of the phospholipid bilayer is the main determinant of the fluidity of biological membranes, and this functionality is the cornerstone of many important physiological functions of cells and organelles. Because we verified that an absorption peak common to various phospholipid bilayers at ~3.0 THz could be assigned to the intrinsic collective vibrations of their similar phospholipid skeletons, we hypothesized that 3.1-THz radiation might enhance the cell membrane fluidity via amplifying this collective vibration of phospholipid bilayers in the cell membrane. RAW264.7 macrophages were used as a cell model, and a classic fluorescence polarization (FP) assay was performed to trial the aforementioned proposition. The FP of N,N,N-trimethyl-4-(6-phenyl-1,3,5,-hexatrien-1-yl) phenylammonium p-toluenesulfonate (TMA-DPH) embedded in the cell membrane is directly related to the micro-fluidity of the cell membrane. The more fluid there is in the cell membrane, the smaller the value of the FP [20]. As shown in Figure 4, the sham group implied that the RAW264.7 macrophages underwent exactly the same treatment as the experimental group except that they were not irradiated by any frequency THz for 20 min, which calibrated the baseline level of the cell membrane fluidity. Exposure to 2.42-THz waves for 20 min was not sufficient to disturb this baseline. However, exposure to 3.1-THz waves for 20 min was adequate to reduce the FP value of TMA-DPH to two-thirds of its original level, suggesting a significant enhancement in the cell membrane fluidity of the RAW264.7 macrophages. It is worth noting that our previous research confirmed that there was no significant change in temperature in the irradiated areas under our experimental conditions, ruling out the possibility of non-specific thermal effects [21].

### 2.5. Improved Phagocytosis of RAW264.7 Macrophages with Irradiation at 3.1 THz

**Figure 5:**
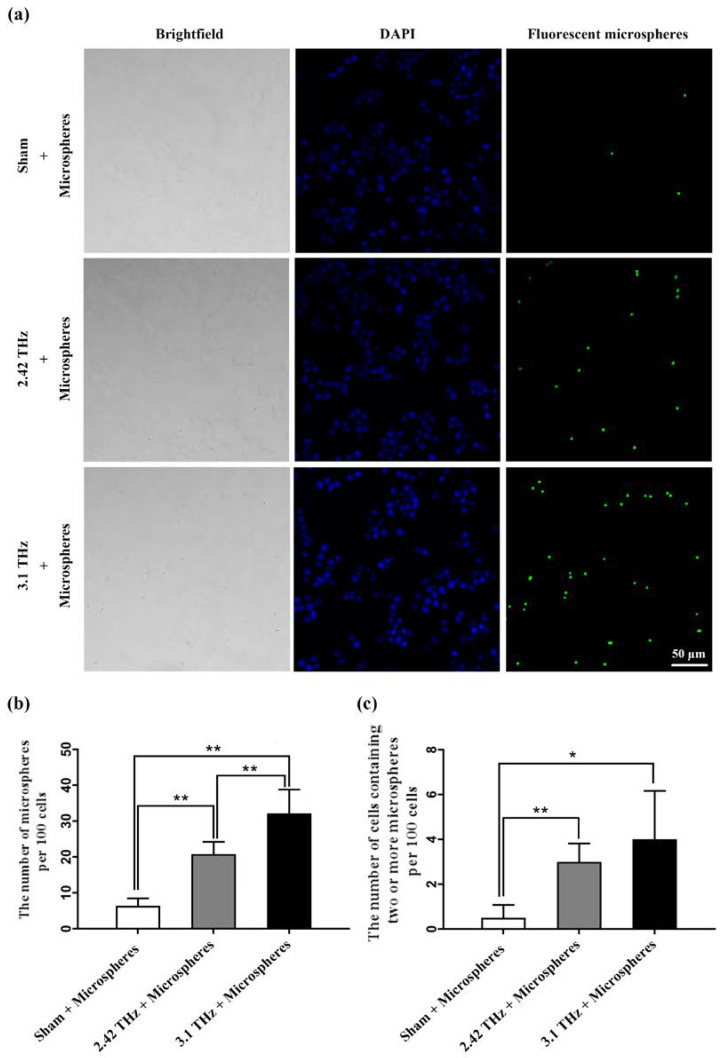
The effect of different frequencies of THz radiation on the phagocytosis of RAW264.7 macrophages. (**a**) LSCM images of RAW264.7 cells incubated with fluorescent microspheres with 2.42, 3.1 THz or without THz irradiation for 20 min. (**b**,**c**) Semiquantitative analysis of the number of intracellular fluorescent microspheres; * *p* < 0.05, ** *p* < 0.01.

Due to the fluidity of the phospholipid bilayers, the cell membranes of macrophages can trap and engulf invading pathogens or apoptotic cells to eliminate them, thereby maintaining bodily homeostasis [22]. Because we observed that 3.1-THz radiation could significantly enhance the cell membrane fluidity, further exploration of its effect on the phagocytosis of RAW264.7 macrophages is helpful for the development of THz biomedical applications. The bright-field and 4′,6-diamidino-2-phenylindole (DAPI) fluorescence images (Figure 5a) demonstrated that the structure of the RAW264.7 cells remained unchanged after irradiation, indicating that no substantial radiation-related toxicity was induced by 20 min of exposure to either 2.42 or 3.1-THz waves. Green-fluorescent microspheres were significantly more abundant in the RAW264.7 cells from the irradiated groups (Figure 5a). A semiquantitative analysis of this image data was consistent with this qualitative observation of the laser scanning confocal microscopy (LSCM) images, indicating that the irradiation at either 2.42 or 3.1-THz resulted in the significantly enhanced phagocytosis of 2.0-μm fluorescent microspheres by RAW264.7 cells. As expected, the 3.1-THz group showed a more vigorous phagocytosis than the 2.42-THz group. The number of fluorescent microspheres per hundred cells in the 3.1-THz group was approximately 7 times that of the sham group and 1.7 times that of the 2.42-THz group (Figure 5b).

## 3. Discussion

Due to the concerted efforts of physicists and biologists in recent years, the biomedical THz spectroscopy field has significantly advanced [23,24,25,26]. However, thus far, the strong THz absorption bands of phospholipid bilayers, which are the primary components of biological membranes, have not been reported because of the lack of suitable THz devices [10,11]. In addition, some studies have theorized that THz exposure may enhance the fluidity of phospholipid bilayers, leading to improved liposome permeability, altered cell morphology, and even repairable or irreparable perforation of the cell membrane [13,14,15]. However, none of these studies have provided a reasonable explanation of the possible mechanisms of these effects or the affected physiological processes wherein phospholipid bilayers play an important role. In this study, we comprehensively explored the spectral characteristics and functional responses of phospholipid bilayers in the THz band using quantum chemical calculations as a “bridge”.

We measured the absorption spectra of the DSPE, DPPC, SPH, and lecithin bilayers in the range of 0.5–4.0 THz at 293 K, which could be used for molecular recognition (Figure 1). The spectra of phospholipid bilayers with the same “hydrophilic head” possessed similar features, regardless of the chain length of the fatty acids and the number of carbon–carbon double bonds. Furthermore, we observed the emergence and blue-shift of the narrow THz absorption peaks of the DSPE and DPPC bilayers as the temperature decreased (Figure 2). This was consistent with the results of studies on other phospholipid bilayers. Moreover, the associated thermodynamic and crystallographic theories have been explained in detail in the literature [7,11]. It is worth noting that, like THz-TDS, inelastic X-ray scattering (IXS) has increasingly been used to study energy-resolved and angle-resolved vibrational patterns in biological materials (including lipid membranes) in recent years [27,28,29]. A combination of THz-TDS and IXS provided compelling evidence for the co-existence of optical and acoustic phonon excitations in mesogenic lipid like systems [30].

Furthermore, we used quantum chemical calculations to demonstrate that the common absorption peak of the DSPE and DPPC bilayers near 3.0 THz originated from an intrinsic collective vibration of the similar long hydrocarbon chains of their “hydrophobic tail” structures (Figure 3). The subtle differences between the theoretically calculated and experimentally measured THz absorption spectra were primarily derived from the limited computational volume of DFT, leading to a lack of thorough consideration of the intermolecular interactions of phospholipids with over 200 atoms.

To investigate the biological effects of THz radiation, we deliberately selected 3.1 THz because we speculated that the exposure of cells to this THz frequency band would enhance the collective vibrations of the phospholipid skeleton. This would result in increased fluidity of the phospholipid bilayer and ultimately cause predictable cell membrane-related functional changes. The FP (Figure 4) and micron-sized fluorescence particle (Figure 5) assay confirmed our hypothesis, demonstrating that 3.1-THz radiation significantly enhanced the cell membrane fluidity and phagocytosis of the RAW264.7 macrophages.

Unfortunately, well-established biology laboratories coupled with the THz free-electron laser device (CTFEL, China Academy of Engineering Physics Terahertz Free Electron Laser, Mianyang, China) used to expose RAW264.7 cells to 3.1-THz radiation are lacking. With the improvement in laboratory construction, further biological studies will be carried out such as the intervention of physiological or pathological processes related to phospholipid bilayer fluidity using 3.1-THz radiation.

## 4. Materials and Methods

### 4.1. Materials

The DSPE, DPPC, SPH, and lecithin (purity ≥ 95%) were purchased from Aladdin (Shanghai, China). COC was kindly donated by Prof. Hongwei Zhao (Shanghai Institute of Applied Physics, Chinese Academy of Sciences, Shanghai, China) and used as a dilutant for the phospholipid bilayer due to its negligible absorption in the THz band [31]. TMA-DPH and FluoSpheres™ microspheres (nominal bead diameter, 2.0 µm; excitation wavelength/emission wavelength, 505/515 nm) were supplied by Sigma-Aldrich (St. Louis, MO, USA) and Invitrogen (San Diego, CA, USA), respectively. Cells from the macrophage-like, Abelson leukemia virus-transformed cell line RAW264.7 were obtained from the National Biomedical Laboratory Cell Resource Bank (Xi’an, Shannxi, China) and cultured according to routine procedures.

### 4.2. Acquisition of Phospholipid THz Absorption Spectra

Samples for the measurement of the THz absorption spectra were prepared using routine procedures [10]. DSPE, DPPC, SPH, and lecithin were dissolved in chloroform, respectively. The solvent was evaporated under reduced pressure for 12 h to remove the organic phase completely. The obtained thin films were split into pieces and uniformly mixed with COC in appropriate ratios. The mixture was subjected to 2000 kg of pressure for 5 s to form a disk with a diameter of 13 mm. The disk was then placed in the sample chamber (filled with dry air at 293 K) of a TAS7400TS THz-TDS system (Advantest, Tokyo, Japan; 1.9 GHz resolution). The absorption coefficients of the samples in the 0.5–4.0 THz range were recorded and used to calculate the corresponding absorption coefficients of phospholipid bilayers according to Equation (1):

(1) $$\alpha_{M}={\;K}_{1}\alpha_{1}+K_{2}\alpha_{2}$$

where α_M_, α_1_, and α_2_ are the absorption coefficients of the mixture, COC, and phospholipid bilayer, respectively, and K_1_ and K_2_ are the mass fractions of the COC and phospholipid bilayer in the mixture, respectively. Origin 8.5 was used to plot the acquired spectra.

### 4.3. Effects of Temperature on DSPE and DPPC THz Absorption Spectra

Liquid nitrogen was injected into the variable-temperature cavity of the sample chamber, and the temperature inside the chamber was monitored in real-time using temperature sensors. When the target temperature (83–293 K) was reached, the absorption coefficients of DSPE and DPPC in the 0.5–4.0 THz range were recorded and calculated as described above. MATLAB R2015a was used to obtain three-dimensional plots showing the temperature dependence of the spectrum.

### 4.4. DFT Calculation of DSPE, DPPC, and SPH Vibrational Frequencies

The optimization of the spatial structures and computation of the vibrational frequencies of DSPE, DPPC and SPH were performed based on DFT using the Gaussian 09 package with the Becke-3 Lee–Yang–Parr functional (B3LYP) [32,33]. The basis sets used for DSPE, DPPC and SPH were 6–31G*, 3–21G*, and 3–21G, respectively, where the 3–21G* basis set includes polarization functions for the P atom only [34,35]. The GaussView 5.0 software was used to export the spectral data. To fit the experimental data, the IR peak half-width at half heights of the DSPE (Figure 3b), DPPC (Figure 3d), and SPH (Figure 3f) used were 0.1, 0.6, and 0.8 THz, respectively.

### 4.5. Influence of Different Frequencies-THz Radiation on Cell Membrane Fluidity of RAW264.7 Macrophages

The CTFEL was used to expose RAW264.7 cells to different frequencies of THz radiation [36]. All of the experiments were conducted at a frequency of 2.42 THz or 3.1 THz, with a pulse width of 2 ms, a peak power per pulse of 1.6 W, and a repetition rate of 10 Hz. The spot diameter was adjusted to 1.1 cm and the resulting average power density was 33 mW/cm^2^. The RAW264.7 cells were seeded at a density of 2 × 10^5^ per coverslip on round coverslips and maintained in a cell incubator (Thermo Scientific, Waltham, MA, USA) at 37 °C for 12 h to ensure attachment. Each coverslip was then placed upside-down in one of the wells of a 24-well plate, and 100 μL of Dulbecco’s modified Eagle’s medium (DMEM) was added into each well to maintain cell survival and minimize THz radiation absorption by water. The cells were cultured in our custom-made thermostatic irradiation incubator at 37 °C, with or without a 20-min dose of 3.1-THz radiation. Subsequently, 1 mL of DMEM containing 2 μM TMA-DPH was then added to each well, and the 24-well plate was transferred to the conventional cell incubator for 15 min. Each coverslip was washed three times with phosphate-buffered saline (PBS) to remove the extracellular TMA-DPH. Finally, the FP of TMA-DPH in the RAW264.7 cells was detected at an excitation wavelength/emission wavelength of 355/430 nm with a multi-function microplate reader (Tecan, Männedorf, Switzerland). FP was calculated automatically by software provided with the instrument, according to Equation (2):

FP = (I_vv_ − GI_vh_)/(I_vv_ + 2GI_vh_) with G = I_hv_/I_hh_, (2)

where I_vv_ and I_vh_ are the intensities of the vertically and horizontally polarized components of the fluorescent light, respectively. G is a grating correction factor for the optical system.

### 4.6. Influence of Different Frequencies-THz Radiation on Phagocytosis by Macrophages

Raw264.7 cells were seeded and irradiated as described above. After with 2.42, 3.1 THz, or without THz irradiation for 20 min, 1 mL of DMEM containing a specific fixed number of fluorescent microspheres was added to each well, and the 24-well plate was transferred to the conventional cell incubator for 15 min. Finally, each coverslip was washed three times with PBS to remove extracellular fluorescent microspheres, and the cells on the coverslip were then subjected to nuclear staining with DAPI before being imaged via LSCM (ZEISS, Jena, Germany).

### 4.7. Statistical Analysis

Three samples of each phospholipid were prepared, and each sample underwent 1028 THz-TDS measurements. All spectra presented in this report were averages. For the macrophage study, all of the experiments were conducted in triplicate, and the data were reported as the mean ± standard deviation values. Differences were considered statistically significant at *p* < 0.05.

## 5. Conclusions

Elucidating the vibrational information encoded within the THz spectrum of biomolecules is critical for guiding the exploration of its functional responses to specific THz radiation wavelengths. To this end, this study explored the THz spectral characteristics of four phospholipid bilayers and demonstrated a common absorption peak around 3.0–3.1 THz that significantly increased the cell membrane fluidity and phagocytosis of RAW264.7 macrophages without any thermal effects. Therefore, irradiation at 3.1 THz is a potential non-invasive strategy to increase the fluidity of lipid bilayers for biomedical applications such as immune activation or drug administration.

## Data Availability

All data that support the findings of this study are included within the article (and any Supplementary Materials).

## Supplementary Materials

The following supporting information can be downloaded at: https://www.mdpi.com/article/10.3390/ijms24087111/s1.
