# Supplementary material for: Spectral Characteristics and Functional Responses of Phospholipid Bilayers in the Terahertz Band
Source: Int J Mol Sci. 2023 Apr 12;24(8):7111. doi: 10.3390/ijms24087111 (PMC10138992; doi:10.3390/ijms24087111)
Supplement: Supplementary file 1 [file ijms-24-07111-s001.zip › ijms-2223753-supplementary.pdf]

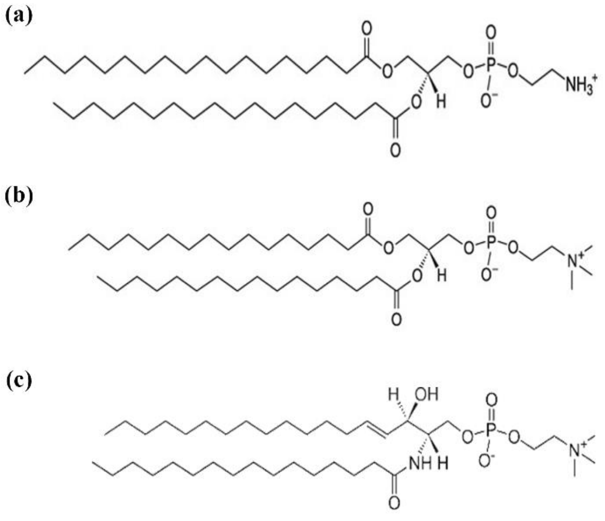

Figure S1. Chemical structures of (**a**) DSPE, (**b**) DPPC, and (**c**) SPH.

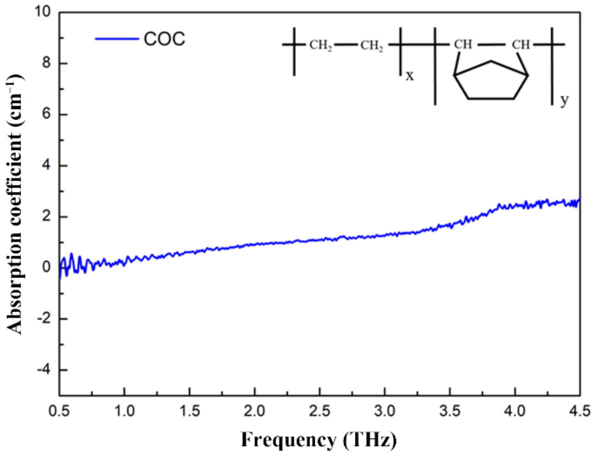

Figure S2. THz absorption spectrum of COC in the 0.5–4.0 THz range at 293 K.

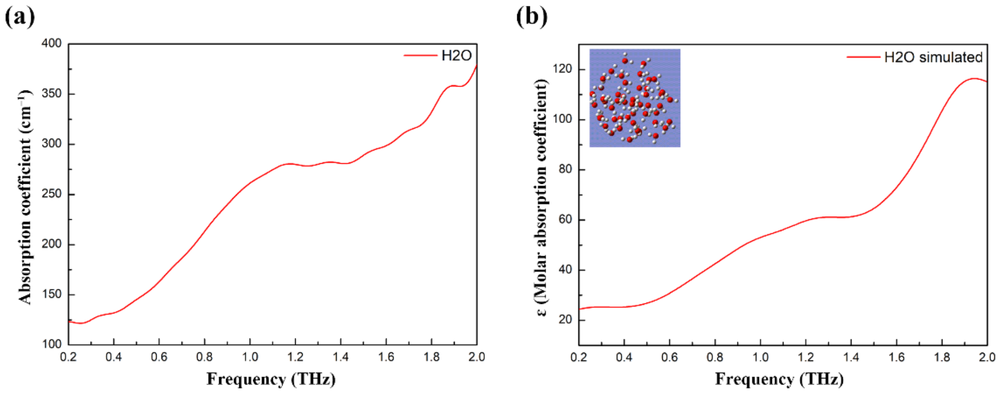

Figure S3. Experimentally and theoretically evaluated THz absorption spectra of H_2O. (**a**) Experimentally measured THz absorption spectra of H_2O at 293 K. (**b**) Theoretically calculated THz absorption spectra of 50 H_2O clusters; the inset shows the predicted molecular structure of the 50 H_2O clusters following geometry optimization.

Table S1. The vibrational modes of DSPE, DPPC, and SPH. (33.33 cm^{-1} = 1 THz)

NUM	Harmonic frequencies (cm^{-1})			IR intensities (KM/Mole)		
	DPPC	DSPE	SPH	DPPC	DSPE	SPH
1	9.19	4.16	2.17	0.4678	0.0273	0.0083
2	13.92	6.63	5.31	0.3144	0.1952	0.9849
3	17.98	8.89	5.65	0.3610	0.3101	1.2340
4	20.14	14.38	9.31	0.3313	0.2093	0.2023
5	23.01	14. 47	13.10	0.5772	0.0083	2.0705
6	27.45	17.63	14.98	0.5273	0.2393	1.1190
7	34.94	19. 85	18.57	0.0643	0.1362	0.1922
8	37.83	23.47	20.18	2.9910	0.1209	0.3571
9	41.20	26.97	21.68	0.0469	0.9497	0.7271
10	42.04	31.87	22.73	0.3107	0.8109	1.3818
11	47.05	35.06	25.70	1.1456	0.7038	0.7152
12	49.02	37.03	28.32	0.2201	1.2139	0.2298
13	54.04	37.40	34.84	0.3775	0.3914	1.6827
14	56.50	39.45	41.96	0.4340	0.5055	0.6308
15	59.15	45.16	46.35	0.2486	0.0089	0.4969
16	63.79	47.73	50.68	0.0900	0.2186	0.0036
17	66.40	50.15	57.65	1.0348	0.4428	0.0069
18	72.64	51.42	62.32	1.9561	1.7507	1.4868
19	75.90	56.09	63.91	0.6375	0.8935	0.1128
20	78.45	58.63	64.66	0.0191	0.3758	1.4731
21	81.43	64.42	69.45	1.5760	1.0291	1.4662
22	88.20	66.37	74.52	1.3155	0.1770	0.2768
23	92.78	69.35	77.80	0.1267	2.1942	0.4305
24	97.55	74.76	84.54	1.0828	3.1492	0.5070
25	99.11	80.51	88.77	0.6001	0.3409	3.6175
26	101.00	82.79	95.25	5.7365	1.3764	1.4820
27	105.05	84.49	98.46	1.6644	1.2524	0.9291
28	108.15	88.88	104.23	1.2388	0.0812	0.7175
29	112.41	93.45	109.31	0.3042	0.1543	1.1896
30	115.75	95.27	111.91	1.6635	0.1441	4.0027
31	118.18	97.22	119.15	0.2592	0.9431	5.3282
32	120.46	100.53	123.02	1.9546	1.1216	1.7221
33	126.03	108.38	123.91	0.2623	0.3852	1.5511
34	127.60	109.75	128.58	0.6832	1.6613	1.7163
35	132.06	116.28	132.14	0.1611	3.5963	0.6261

36	134.81	120.78	140.86	0.2211	0.0940	0.5613
37	140.59	125.31	144.04	0.7458	0.0765	2.3715
38	148.26	125.71	144.99	0.2240	0.1690	1.5553
